# Invasive Mole Mimicking Abnormal Uterine Bleeding: A Case Report

**DOI:** 10.7759/cureus.35195

**Published:** 2023-02-19

**Authors:** Darshan D Sankhe, Savita Somalwar, Farah Jiandani, Sheela Jain, Anushree Shetty

**Affiliations:** 1 Department of Obstetrics and Gynaecology, Narendra Kumar Prasadrao (NKP) Salve Institute of Medical Sciences and Research Centre, Nagpur, IND

**Keywords:** chemotherapy, human chorionic gonadotropin, abdominal hysterectomy, invasive mole, gestational trophoblastic disease

## Abstract

Invasive mole (IM) is a very uncommon subtype of gestational trophoblastic disease (GTD), which is the invasion of molar tissue into the uterine or myometrial vasculature. However, this report presents a rare case of a 41-year-old female multiparous P7 with five full-term normal vaginal deliveries and two preterm normal vaginal deliveries. As the patient was not using contraception, her urine pregnancy test (UPT) was done, which demonstrated positive results. A speculum examination revealed a healthy cervix with just mild bleeding, whereas a vaginal examination revealed a firm cervix and an anteverted and mobile eight-week-old uterus along with a free fornix. Pelvic ultrasound and magnetic resonance imaging (MRI) demonstrated the diagnosis of GTD, for which consultation from an oncology physician was taken and the treatment proceeded with a total abdominal hysterectomy. Histological examination of the uterus showed a circumscribed nodule showing a large area of hemorrhage with few chorionic villi lined with trophoblastic cells and occasional villi invading the myometrium and endometrial cavity suggesting hydatidiform mole showing early invasion that confirmed the diagnosis of IM. In conclusion, reproductive-age women who experience abnormal uterine bleeding (AUB) should suspect pregnancy with several possible complications, for which a pregnancy test should be done to rule out complications.

## Introduction

Invasive moles (IMs), a rare subset of gestational trophoblastic disease (GTD), are molar tissue consisting of hydropic villi with hyperplasia of the trophoblastic elements and can invade the myometrium or uterine vessels in women of childbearing age [1,2]. The first instance of IM was reported in Madagascar in 1965, and a new case was identified in 2018 [1,3]. Invasion of the myometrium by edematous villi and proliferative trophoblasts allows them to be differentiated from choriocarcinoma. Villi are reported to be present in trophoblastic tissue by pathologists [4,5]. In persistent gestational trophoblastic tumors, when the antecedent pregnancy is partial, the hydatidiform mole can have malignant sequelae [1].

Localized invasive gestational trophoblastic neoplasia (GTN) develops in 15% of people after total mole excision and sporadically after partial mole removal. It becomes a metastatic condition in 4% of patients [6]. Human chorionic gonadotropin (HCG) concentrations (>100,000 mIU/mL), abnormal uterine development, and theca lutein cysts (6 cm) are associated with high risks for developing post-molar malignancies [7]. The most typical indication of an IM is persistent vaginal bleeding after the molar pregnancy has been eliminated. The increase in beta-HCG titer is a laboratory test used to identify an IM during monitoring and follow-up of molar pregnancy. An IM can be definitively diagnosed using histology; however, it can also be found via HCG or radiological techniques [4,8].

The most frequent site for metastasis is the lungs (80%), followed by the vagina (30%), pelvis (20%), liver (10%), brain (10%), and bowel, kidney, and spleen (<5%) [2]. Ovarian metastasis from an IM is rare, and about 5%-6% of ovarian cancers result from other organ metastasis. Metastasis can occur as a direct extension of another pelvic tumor, a hematogenous or lymphatic spread, or a transcoelomic dissemination. The probability of metastasis to the ovary is even less likely than the incidence of non-gestational primary ovarian choriocarcinoma, which is one in 3.7 × 10^8^ [2,9]. Chemotherapy (CT) can treat IM, but numerous chemotherapy sessions are unnecessary after a hysterectomy. It is still required in patients with severe bleeding or sepsis for complication control and stabilization [2,10].

## Case presentation

A 41-year-old multiparous female, married with P7L5D2, presented with chief complaints of continuous bleeding per vagina and occasional passage of clots since the last menstrual cycle 45 days ago, for which she had received symptomatic treatment in a private hospital with no further investigations or relief. Additionally, the patient complained of fatigue, dizziness, shortness of breath, anxiety or irritability, sleep problems, and unexplained weight loss. There was no significant past medical or surgical history, and she was not using any contraceptive method.

On examination, the build of the patient was found to be average with a weight of 65 kg and a body mass index (BMI) of 22.5 kg/m². The patient was afebrile, and all vitals were within normal limits: heart rate, 78 beats per minute; respiratory rate, 19 breaths per minute; blood pressure, 150/90 mmHg; and peripheral capillary oxygen saturation (SpO2), 96%. The local genital examination was normal with no discoloration, swelling, or discharge. The cervix was healthy with minimal bleeding present per speculum examination, and the cervix was firm on vaginal examination, with a uterus of eight weeks that was anteverted and mobile along with a free fornix.

On investigations that included complete blood count and biochemical evaluation, the total leukocyte count was 6,820 cells/mm^3 ^and the platelet count was 1.87 lakhs/mm^3^ with reduced hemoglobin (Hb) percentage of 11.05 mg%. Considering the patient’s age and lack of contraception, a urine pregnancy test (UPT) was done, which was found to be positive. The patient was admitted with a provisional diagnosis of P7L5D2 with abnormal uterine bleeding (AUB) under evaluation.

**Figure 1:**
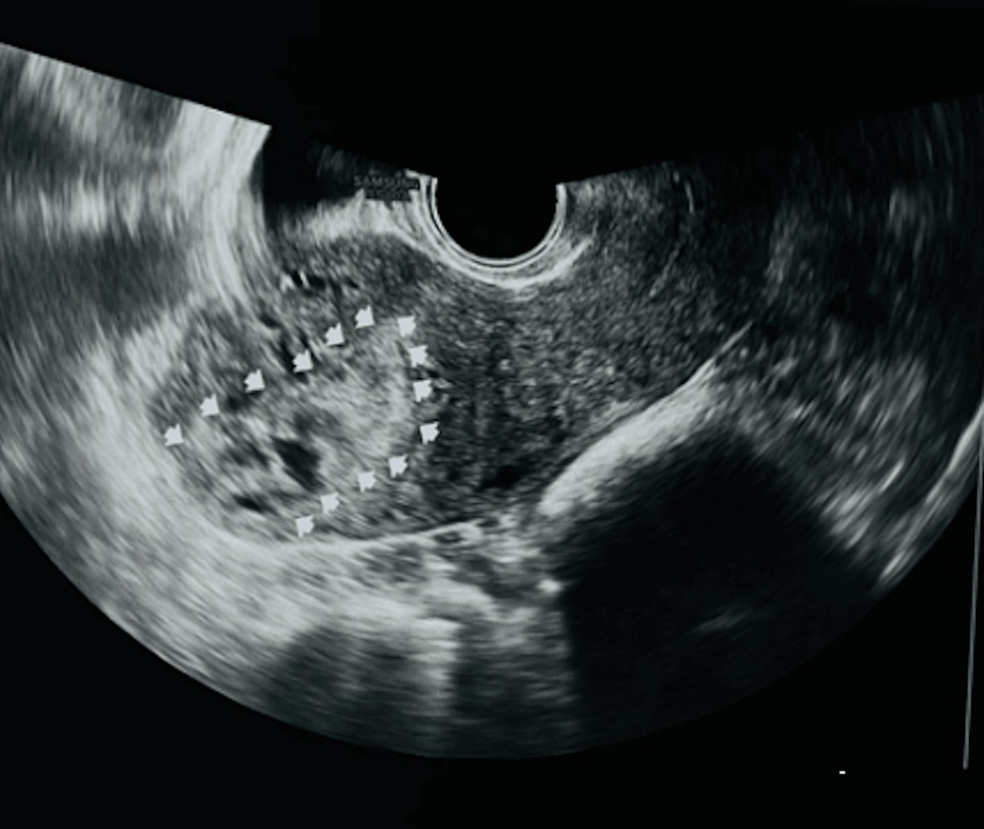
Ultrasonography showing the intra-myometrial mass

The diagnostic assessment that involved ultrasonography (USG) revealed a bulky uterus of 9 × 6 × 5 cm, with a heterogenous lesion, predominantly hyperechoic, measuring 2.8 × 3.4 × 2 cm, with no evidence of calcification. Vascularity on color Doppler was present with vascular channels from the myometrium supplying the lesion noted. The USG impression was changes of adenomyosis with a heterogenous lesion in the fundus and heterogenous endometrial thickness (ET) (Figure 1).

With a confusing picture of USG and a positive UPT, beta-HCG was done to confirm pregnancy, which showed increased values (18,097.6 IU/mL), following which magnetic resonance imaging (MRI) was done that revealed a bulky uterus measuring 9.8 × 6.9 × 5.9 cm, which was heterogenous in intensity with normal ovaries along with an ill-defined altered enhancing lesion measuring 3.5 × 3.3 × 4.2 cm noted in the endometrium and in the fundal area extending into the anterior myometrium. The anterior myometrium measured 3.7 cm, and >50% anterior myometrium was replaced by multiple dilated vascular channels. Hence, the probable diagnosis based on the above findings, along with increased beta-HCG and UPT positive status, was suggestive of GTN with hydatidiform mole showing early invasion leading to IM.

**Figure 2:**
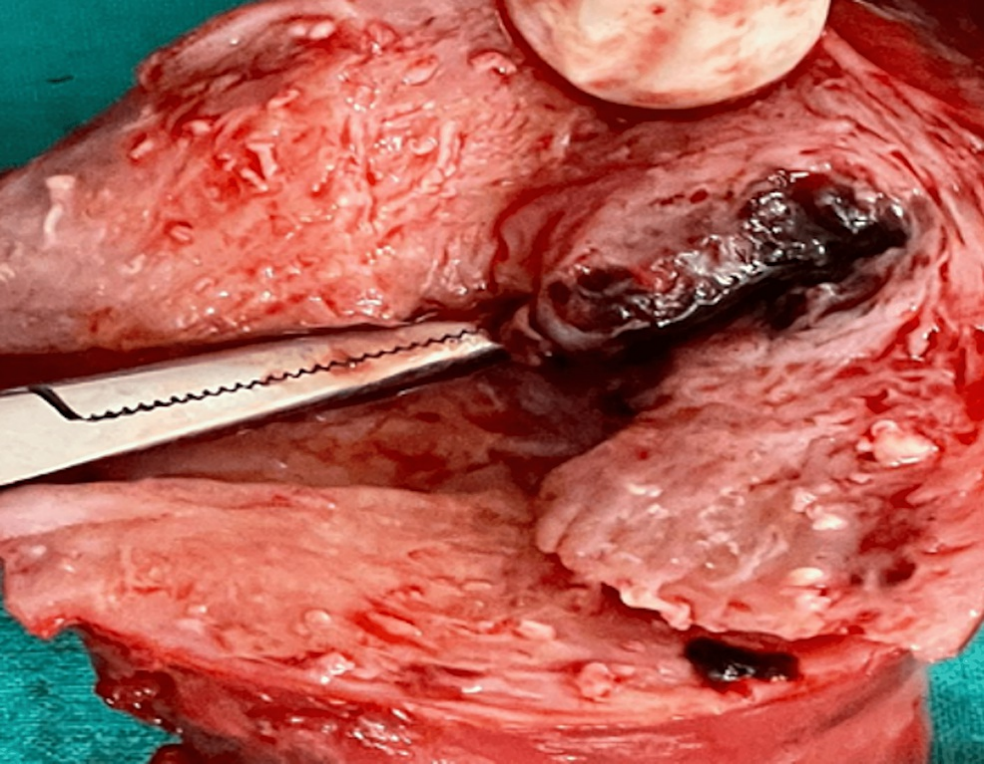
Intraoperatively visible invasive mole

Following a discussion with the oncologist, the decision on total abdominal hysterectomy with bilateral salpingo-oophorectomy was taken. The purpose of this case report was explained to the patient, and written informed consent was obtained before commencing the surgical intervention. Intraoperatively, a bluish lesion of 3 × 3 × 2 cm was present on the anterior and fundal wall of the uterus, and the lesion from the endometrium was extending into the myometrium. There were areas of necrosis and hemorrhage. Figure 2 shows the IM intraoperatively.

**Figure 3:**
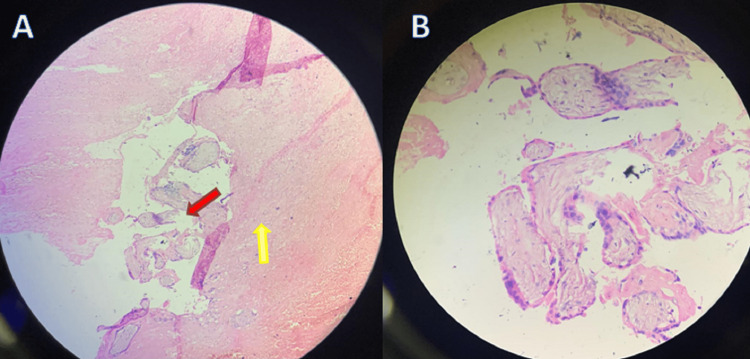
Histopathology slide showing villi encroaching the myometrium where the red arrow highlights the villi and the yellow arrow highlights the myometrium (A) and high-power field focusing on the villi (B)

Furthermore, the histopathology report of the uterus showed round to oval endometrial glands, cystically dilated and lined with tall columnar cells, a stroma composed of spindle-shaped cells, and a circumscribed nodule showing a large area of hemorrhage with few chorionic villi as demonstrated in Figure 3A and Figure 3B. The villi were hydropic or fibrotic, avascular, and lined by trophoblastic cells, and the peripheral area showed a rim of decidual cells. Occasional villi were seen invading the myometrium, suggestive of a hydatidiform mole showing early invasion.

According to the modified World Health Organization (WHO) prognostic scoring for GTN, the final diagnosis of IM stage I with a prognostic score of 9 was confirmed. The postoperative course was uneventful, and on postoperative day 4, beta-HCG was repeated, which was <2 IU/mL. The patient was discharged with close follow-up and monitoring of beta-HCG.

## Discussion

This case report highlights a rare case of invasive mole (IM) stage I with a prognostic score of 9 along with the effectiveness and adherence of the patient to surgical intervention that mainly involved total abdominal hysterectomy. IM belongs to the GTD histological subgroup. Choriocarcinoma, placental site trophoblastic tumor (PSTT), and epithelioid trophoblastic tumor are the other three varieties of GTD [3,11]. Although its incidence is not well known, less than 1% of all gynecological tumors are GTD [12]. It usually follows a molar pregnancy [13], but regardless of its results, it can occur after any pregnancy [2,3]. To prevent delay in the diagnosis, a histological investigation of all abortion products is therefore indicated.

Pelvic USG and HCG dosage serum confirm the diagnosis. Histology, however, provides conclusive confirmation of an IM [3,4,14]. In this instance, the diagnosis of an IM was not made until the surgical specimen had undergone a histological analysis. The gold standard for IM diagnosis is USG before surgical intervention [15]. An atypical uterine mass can be found with the help of B-mode USG. IM, implantation site tumors, and choriocarcinomas typically appear on USG as heterogenous, hyperechogenic solid masses in the myometrium with cystic vascular gaps [16].

In cases of abdominal urgency caused by uterine bleeding, severe bleeding, sepsis, and the patient who had finished her parental project, a hysterectomy is recommended as a specialized form of treatment to manage difficulties and stabilize patients [2,10,17,18]. According to the International Federation of Gynaecology and Obstetrics (FIGO) and the WHO prognosis classification category, CT is currently the primary treatment for IM and is effective in almost 100% of instances [3]. Starting with the first two weeks of consecutively negative beta-HCG levels, a follow-up of at least 12 months with monthly beta-HCG level determination is advised [1].

## Conclusions

This case report concludes that, as IM is a rare GTD that can be life-threatening, reproductive-age women who experience abnormal uterine bleeding (AUB) should suspect pregnancy with several possible complications, for which a pregnancy test should be done to rule out complications. A histological examination establishes the diagnosis of these uncommon pathologies, and interdisciplinary management aids in improving the prognosis. Early detection enables the commencement of CT, allowing for the prevention of consequences and the preservation of the obstetric prognosis. A hysterectomy may be an option when the essential prognosis is at risk.
